# Predictive Value of Peri-Coronary Fat Attenuation Index in Elderly Non-ST-Elevation Myocardial Infarction and Its Correlation with Platelet to Lymphocyte Ratio

**DOI:** 10.1186/s12872-025-04730-8

**Published:** 2025-04-18

**Authors:** Yanglei Li, Chen Wang, Qinyue Wang, Siqi Li, Junchao Yang, Hanqin Pan, Ming Li, Xinkai Qu, Yiran Qin, Chunwei Ma, Lin Qi, Mingxuan Li, Kailei Shi

**Affiliations:** 1 Department of Cardiology, Huadong Hospital, Fudan University, Shanghai, China; 2 Department of Gastrointestinal Endoscopy, Huadong Hospital, Fudan University, Shanghai, China; 3 Department of Radiology, Huadong Hospital, Fudan University, Shanghai, China; 4 Department of Information Technology, Huadong Hospital, Fudan University, Shanghai, China; 5 Department of Cardiology, Qingpu Branch of Zhongshan Hospital Affiliated to Fudan University, Shanghai, China; 6 Department of Genaral Practice, Zhongnan Hospital of Wuhan University, Hubei, China

**Keywords:** Peri-coronary adipose tissue, Fat attenuation index, Platelet to lymphocyte ratio, Non-ST-elevation myocardial infarction, Elderly

## Abstract

**Background:**

Inflammatory mediators and immune activation can intensify the inflammatory response within atherosclerotic plaques, increasing the risk of plaque rupture and thrombosis. This study aims to compare peri-coronary adipose tissue fat attenuation index (PCAT-FAI) and platelet-to-lymphocyte ratio (PLR) between NSTEMI and SA patients, and explore their combined predictive ability for NSTEMI.

**Patients and methods:**

: The study included 52 NSTEMI patients aged 65 and older who underwent both CCTA and CAG from January 2020 to December 2022, alongside 52 gender- and age-matched SA patients, and 52 control patients. PCAT-volume and PCAT-FAI were measured using Siemens VB20.0, and PLR was calculated from peripheral blood tests.

**Results:**

Among 156 patients, NSTEMI patients had significantly higher PCAT volume (12.13 ± 5.32 mm³) compared to SA (8.39 ± 4.10 mm³, *p* < 0.001) and controls (6.28 ± 3.40 mm³, *p* < 0.001). They also had higher PCAT-FAI (-76.28 ± 5.33 HU) than SA (-82.87 ± 6.19 HU, *p* < 0.001) and controls (-84.19 ± 5.74 HU, *p* < 0.001). PLR was higher in NSTEMI patients (178.27 ± 107.18) compared to SA (115.54 ± 45.28, *p* = 0.002) and controls (116.09 ± 38.09, *p* = 0.006), with no significant difference between SA and controls (*p* = 1.000). PCAT-FAI correlated with PLR (CC: 0.298; *P* < 0.01). Combining PLR and PCAT-FAI predicted NSTEMI with an AUC of 0.799 (95% CI, 0.715–0.883).

**Conclusion:**

Higher PCAT-FAI and PLR in NSTEMI patients highlight the role of adipose tissue inflammation and thrombosis in coronary artery disease progression. Combined assessment of PCAT-FAI and PLR has potential value in predicting the adverse progression of atherosclerotic plaques.

## Background

The majority of non-ST elevation myocardial infarction (NSTEMI) incidents are found in older adults [1]. The proportion of the global population aged 80 years or older is projected to triple over the next 20 years. Advancing age is a key predictor of negative events in patients with this coronary artery disease [2]. In NSTEMI, a coronary artery experiences partial obstruction due to a thrombus, leading to decreased blood flow, unlike the full blockage seen in ST-elevation myocardial infarction (STEMI) [3]. This condition causes ischemia and damages heart muscle without the ST-segment elevation typically observed in STEMI on an electrocardiography (ECG). Additionally, elderly patients often lack the classic symptoms of chest pain, making the diagnosis of NSTEMI more challenging. Delays in diagnosis can further lead to untimely treatment, resulting in poor prognosis for the patient. In recent times, there has been a focus on exploring a wide array of inflammatory indicators with the goal of discovering novel biomarkers. These efforts are directed towards enabling the early and precise identification of individuals at elevated risk for cardiovascular diseases (CVD).

Research spanning both preclinical and clinical domains has unveiled that inflammation and metabolic disturbances play a pivotal role at every stage of atherosclerosis, significantly contributing to the instability of plaques and the disease’s advancement [4–6]. Over the past several decades, various inflammatory and metabolic biomarkers have been studied, including the roles of the inflammatory cytokine C-reactive protein (CRP) and the pro-inflammatory cytokine Interleukin-6 (IL-6) in patients with CVD [7–10]. However, these traditional biological markers, which are more reflective of the body’s inflammation and immune status, have relatively low specificity, rendering them less effective for the rapid diagnosis of acute myocardial infarction.

The Fat Attenuation Index (FAI) is a quantitative metric used in medical imaging, particularly in computed tomography (CT) scans, to assess the attenuation or density of adipose tissue [11]. Under inflammatory conditions, changes in the distribution and quality of adipose tissue may occur, and increased FAI values may indicate alterations in the inflammatory status of adipose tissue, potentially reflecting the presence of inflammatory processes or diseases [12, 13]. Early studies have established a connection between elevated peri-coronary adipose tissue (PCAT) FAI, assessed through Coronary Computed Tomography Angiography (CCTA), and increased levels of coronary artery inflammation, leading to an elevated risk of coronary artery disease (CAD) [14, 15]. Further studies have discovered that CT imaging of PCAT inflammation can specifically identify high-risk plaques (HRP) across a spectrum from stable CAD to acute coronary syndrome (ACS), with attenuation levels gradually increasing and being independent of standard circulating inflammatory biomarker [16, 17].

The novel biomarker platelet to lymphocyte ratio (PLR) discovered in recent years, not only mirror the body’s inflammation and immune status but also more accurately reflect the body’s thrombotic and pre-thrombotic conditions [18, 19]. As a result, it is increasingly used to monitor the progression of cardiovascular disease [20]. However, there is no research on the correlation between PLR and adipose tissue inflammation. And so far, the utility of PCAT-FAI in conjunction with PLR to identify NSTEMI has not been established.

This study involved contrasting PCAT-FAI and PLR variances between patients with NSTEMI and stable angina (SA), while also investigating the correlation between PCAT-FAI and the emerging biomarkers PLR. Furthermore, we assessed the capability of PCAT-FAI when combined with PLR for predicting NSTEMI.

## Materials and methods

### Study population

This is a retrospective clinical study that included 52 patients aged 65 years or older, seeking treatment at the Chest Pain Center of Huadong Hospital between January 2020 and December 2022.These individuals presented with chest pain, underwent CCTA scans, and were diagnosed with NSTEMI based on emergency ECG, cardiac enzyme analysis, and subsequent coronary angiography (CAG). Exclusion criteria comprised the following: STEMI, a prior history of myocardial infarction or revascularization, ongoing acute infection, severe autoimmune diseases, or prolonged corticosteroid usage, concurrent presence of tumors, incomplete clinical data, and ambiguous or absent image sequences.

During the same period, 52 well-matched patients were enrolled in the SA group, who sought cardiovascular clinic, underwent CCTA scans, and were diagnosed with SA through ECG, cardiac enzymes, and subsequent CAG. Matching was performed based on age and gender using propensity score matching (PSM).

Furthermore, an additional 52 elderly patients, who underwent outpatient CCTA examinations and exhibited either no coronary artery stenosis or stenosis less than 25% during CAG, were assigned as the control group.

**Fig. 1:**
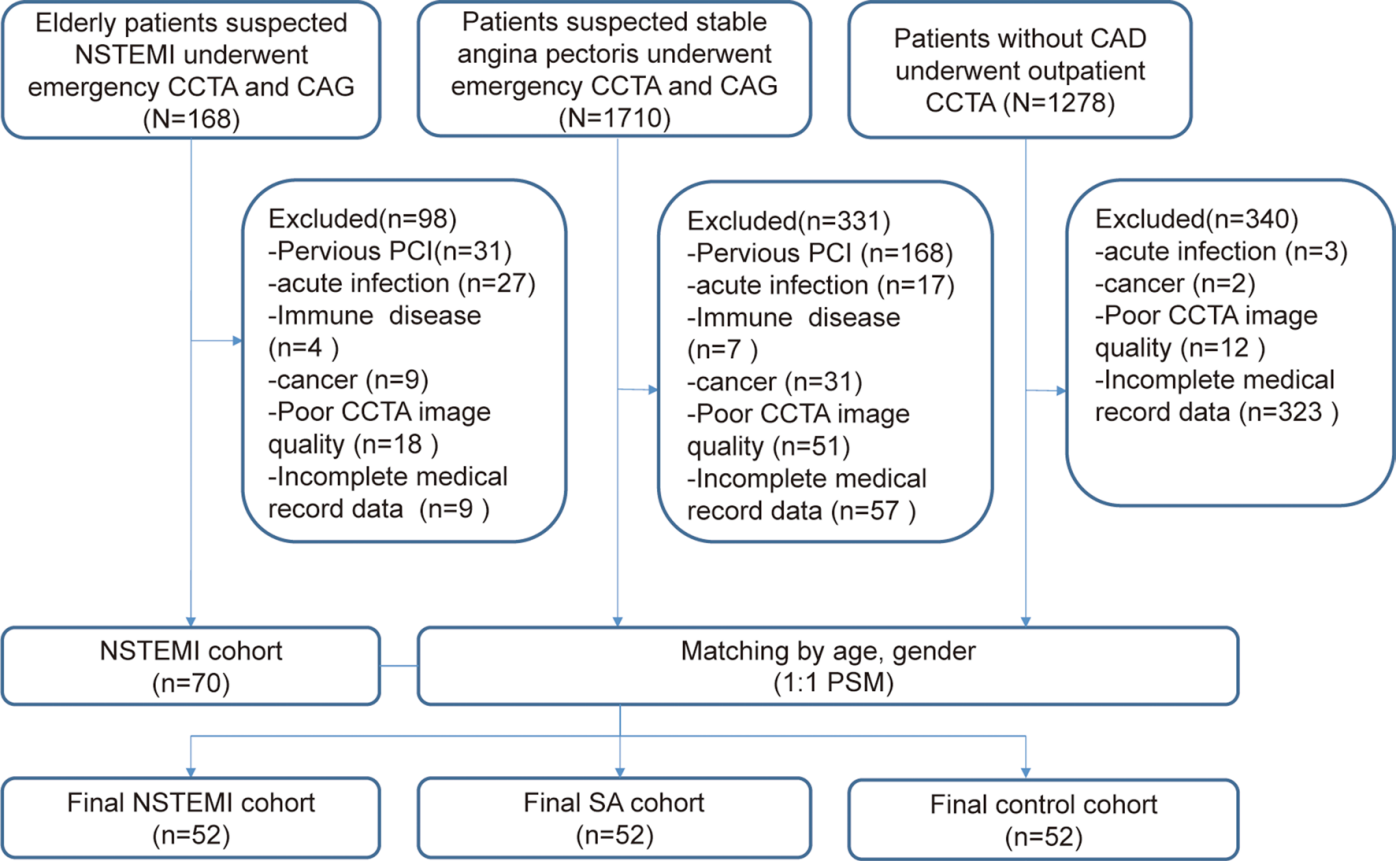
Flowchart. NSTMEI, non-ST segment elevation myocardial infarction; CCTA, coronary computed tomography angiography; CAG, coronary angiography; PCI, percutaneous coronary intervention; CAD, Coronary artery disease: SA, stable angina; PSM, Propensity Score Matching

Figure 1 illustrates the study design and the process of patient selection. This study was performed in accordance with the Declaration of Helsinki. Before the study began, we had obtained informed consent from all subjects. Ethical approval for this research has been granted by Huadong Hospital ethics committee (clinical trial number: 2019K072).

### CCTA protocol

Participants underwent CCTA scans utilizing a Siemens CT scanner (Siemens, Definition Flash, Erlangen, Germany) under the guidance of skilled technicians, adhering to a standardized protocol. The CT scan encompassed the area from the tracheal bifurcation to the apex of the heart. Scan parameters comprised detector collimation (256 × 0.625 mm), reconstruction slice thickness (0.625 mm), slice interval (0.625 mm), gantry rotation time (0.28 s), tube voltage (100 kV), and smart-mA adjustment based on the patient’s condition. The field of view (FOV) was set at 25 cm.

All participants had an 18G intravenous catheter placed in the upper limb vein. The contrast agent, iopromide (370 mg iodine/mL, Bracco, Italy), was injected at a rate of 4.5 to 5.5 mL/s, adjusted according to their body mass index (BMI) and venous condition, with a total volume ranging from 50 to 60 ml. Following the contrast agent injection, a flush of 30 to 40 mL of normal saline was administered at the same injection rate.

### Analysis of PCAT volume and Attenuation

The PCAT-FAI was measured using a Siemens VB20.0 workstation and post-processing software. Image reconstruction for PCAT measurement was performed at the 80% point of the R-R interval. The coronary arteries of interest were manually selected, and the heart was separated to remove the blood pool, retaining only the coronary artery tree. A segmentation tool was used to fine-tune the region of interest (ROI) around the coronary artery tree, with a fat threshold set between − 190 HU and − 30 HU. After the evaluation, quantitative data of the attenuation index and volume of PCAT surrounding the coronary artery tree within the ROI were obtained. The PCAT volume represented the total peri-coronary adipose tissue volume around the left anterior descending artery (LAD), left circumflex artery (LCX), and right coronary artery (RCA), while PCAT-FAI represented the average attenuation index of adipose tissue around the LAD, LCX, and RCA. All PCAT-FAI parameters were measured by two independent investigators blinded to clinical information. In cases of high discordance, measurements were repeated. The correlation between intra- and interobserver for PCAT volume was 0.928 (*p* < 0.001), and for PCAT-FAI, it was 0.959 (*p* < 0.001).

### Measurement of PLR

Blood samples from NSTEMI patients who arrived at the emergency department were drawn from the median cubital vein upon their arrival. Meanwhile, for patients admitted to the hospital, samples were obtained through the median cubital vein within one hour of their admission. These specimens were then stored in Ethylenediaminetetraacetic acid tubes for a full blood cell count, conducted with an automated blood analysis machine. The PLR was calculated by dividing the total platelet count by the absolute count of lymphocytes.

### Statistical analysis

All data underwent statistical analysis using SPSS 23.0. The normality of variables was assessed with the Shapiro–Wilk test. Normally distributed continuous variables were expressed as mean ± standard deviation, while non-normally distributed continuous variables were presented as median (interquartile range). Categorical data were represented as frequency and percentage [n (%)]. Categorical data among groups were compared using either the Pearson’s chi-square test or Fisher’s exact chi-square test. Unpaired t-test or Mann–Whitney U test was used for comparing continuous variables. To compare measurement data across more than three groups, we applied analysis of variance (ANOVA), followed by post-hoc comparisons using independent samples t-tests. Spearman’s correlation test was employed to analyze associations among measurement data. Logistic regression analysis strategy followed a systematic, stepwise methodology. Initially, we performed univariable logistic regression analysis on all candidate variables. Subsequently, we conducted multivariable logistic regression analysis, adjusting for established cardiovascular risk factors including hypertension, diabetes mellitus (DM), and smoking status, along with other variables that demonstrated statistical significance (*P* < 0.05) in the univariable analysis. Receiver Operating Characteristic (ROC) curves were used to evaluate diagnostic accuracy and establish threshold points. A significance level of *P* < 0.05 was deemed statistically significant.

## Results

### Participants characteristics

The clinical characteristics and biochemical parameters of the participants are shown in Table 1. There were no statistically significant differences across the groups with regard to age, gender, body mass index, medical history, triglycerides and low-density lipoprotein. The smoking rate in the NSTEMI or SA group was higher than that in the control group, but there was no significant difference between the NSTEMI and SA groups. Additionally, in terms of lipid metabolism, patients in the SA group had lower levels of total cholesterol compared to the other two groups, with the control group having higher levels of high-density lipoprotein than the NSTEMI group.

**Table 1:** The clinical characteristics and biochemical parameters of the participants

Characteristic	NSTEM(*n* = 52)	SA(*n* = 52)	Controls(*n* = 52)	*p* value
Demographics				
Age (years)	71.23 ± 5.54	73.13 ± 6.77	71.19 ± 5.69	0.138
Male, n (%)	39(75.0%)	36(69.2%)	31(59.6%)	0.236
BMI (kg/m^2^)	25.20 ± 3.56	24.37 ± 4.27	24.29 ± 3.14	0.388
Clinical risk factors				
Hypertension, n (%)	39(75.0%)	40(76.9%)	35(67.3%)	0.504
Diebetes mellitus, n (%)	25(48.1%)	26(50.0%)	22(42.3%)	0.716
Stoke. n (%)	3(5.8%)	9(17.3%)	4(7.7%)	0.115
Atrial fibrillation, n (%)	3(5.8%)	4(7.7%)	5(9.6%)	0.763
Smoking. n (%)	20(38.5%)	16(30.8%)	7(13.5%)	0.014
Laboratory values				
Total cholesterol (mmol/L)	4.43[3.71–4.99]	3.98[3.48–4.70]	4.66[4.22–4.99]	0.008
Triglyceride (mmol/L)	1.45[1.16–1.99]	1.40[1.03–2.12]	1.76[1.07–2.36]	0.312
HDLc (mmol/L)	1.17[1.01–1.29]	1.25[1.03–1.39]	1.37[1.09–1.55]	0.017
LDLc (mmol/L)	2.56[2.00-3.14]	2.34[1.79–2.99]	2.66[2.41–3.12]	0.137
CRP(mg/L)	4.62[1.47–26.80]	2.34[1.22–5.63]	1.98[1.04–3.99]	0.001
cTnT(ng/ml)	0.53[0.18–2.09]	0.01[0.01–0.02]	0.01[0.00-0.01]	<0.001
GENSINI score	73.50[46.00–91.00]	9.00[5.00–25.00]	0.00[0.00–0.00]	<0.001

NSTEMI, non-ST elevation myocardial infarction; SA, stable angina; BMI, body mass index; HDLc, high density lipoprotein cholesterol; LDLc, low density lipoprotein cholesterol; CRP, c-reactive protein; cTNT, cardiac Troponin T

### PCAT volume and PCAT-FAI among three groups

**Fig. 2:**
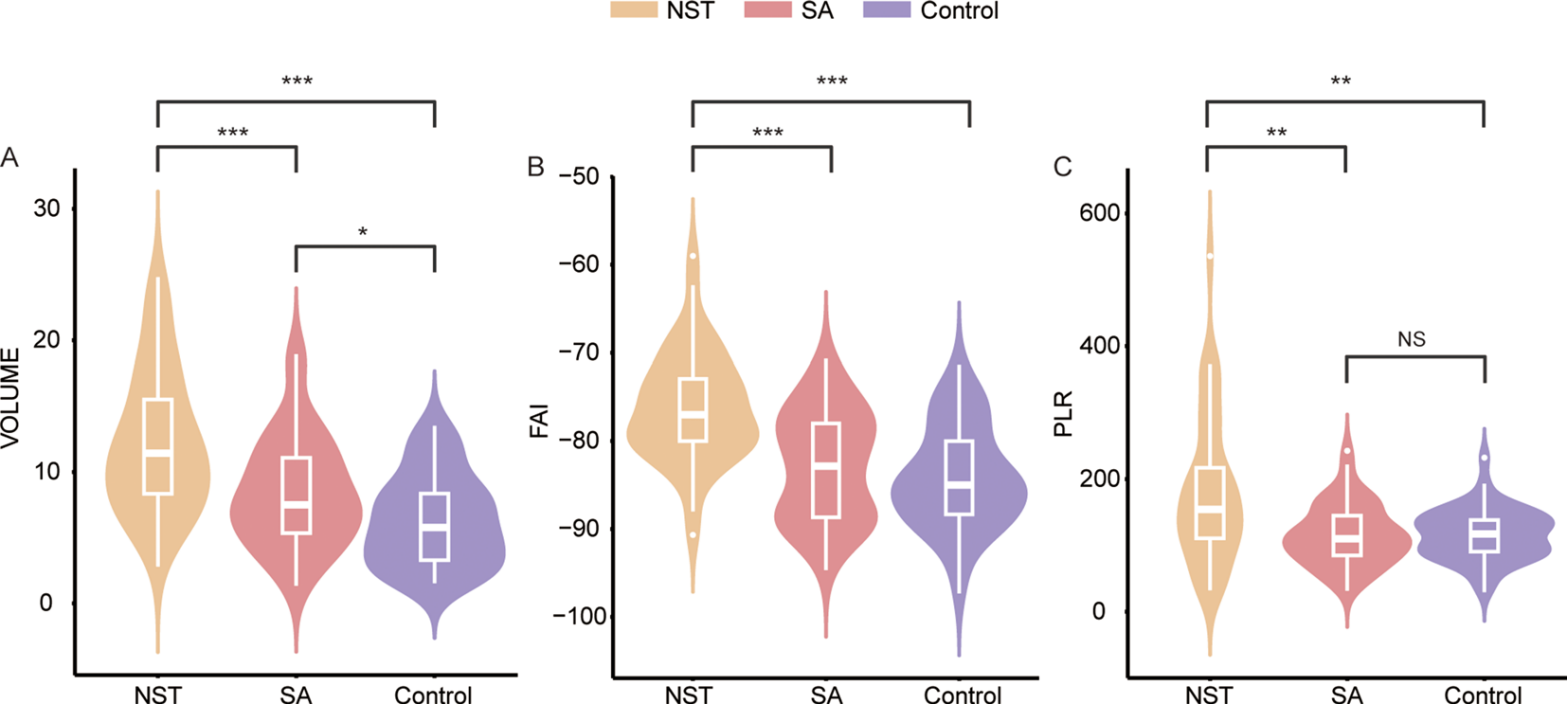
PCAT volume, PCAT-FAI and PLR among three groups. (**A**) The PCAT volume in the NST group was higher than SA and control group, and the SA group was higher than control group; (**B**) The PCAT-FAI in the NST group was higher than SA and control group; (**C**) The PLR in the NST group was higher than SA and control group. NSTMEI, non-ST segment elevation myocardial infarction; SA, stable angina; PLR, platelet to lymphocyte ratio; PCAT, peri-coronary adipose tissue; FAI, fat Attenuation Index

The volume of peri-coronary adipose tissue exhibited statistically significant differences among the NSTEMI group, SA group, and control group (*p* < 0.001). The PCAT volume (12.13 ± 5.32 mm³) in NSTEMI patients was significantly higher than that in the SA group (8.39 ± 4.10 mm³, *p* < 0.001) and the control group (6.28 ± 3.40 mm³, *p* < 0.001). Additionally, the PCAT volume in the SA group was also higher than that in the control group (*p* = 0.014). (Fig. 2A) The PCAT-FAI values progressively increased in the control group, SA group, and NSTEMI group (*p* < 0.01). The PCAT-FAI in NSTEMI patients (-76.28 ± 5.33 HU) was significantly higher than that in the SA group (-82.87 ± 6.19 HU, *p* < 0.001) and the control group (-84.19 ± 5.74 HU, *p* < 0.001). However, the difference in PCAT-FAI between the SA group and the control group did not reach statistical significance (*p* = 0.264) (Fig. 2B).

### Platelet to lymphocyte ratio among three groups

Patients in the NSTEMI group (178.27 ± 107.18) showed significantly higher PLR values compared to the SA group (115.54 ± 45.28, *p* = 0.002) and the control group (116.09 ± 38.09, *p* = 0.006), with no statistical difference in PLR values between the SA group and the control group (*p* = 1.000) (Fig. 2C).

### Correlation between PCAT-FAI and PCAT volume with PLR

**Fig. 3:**
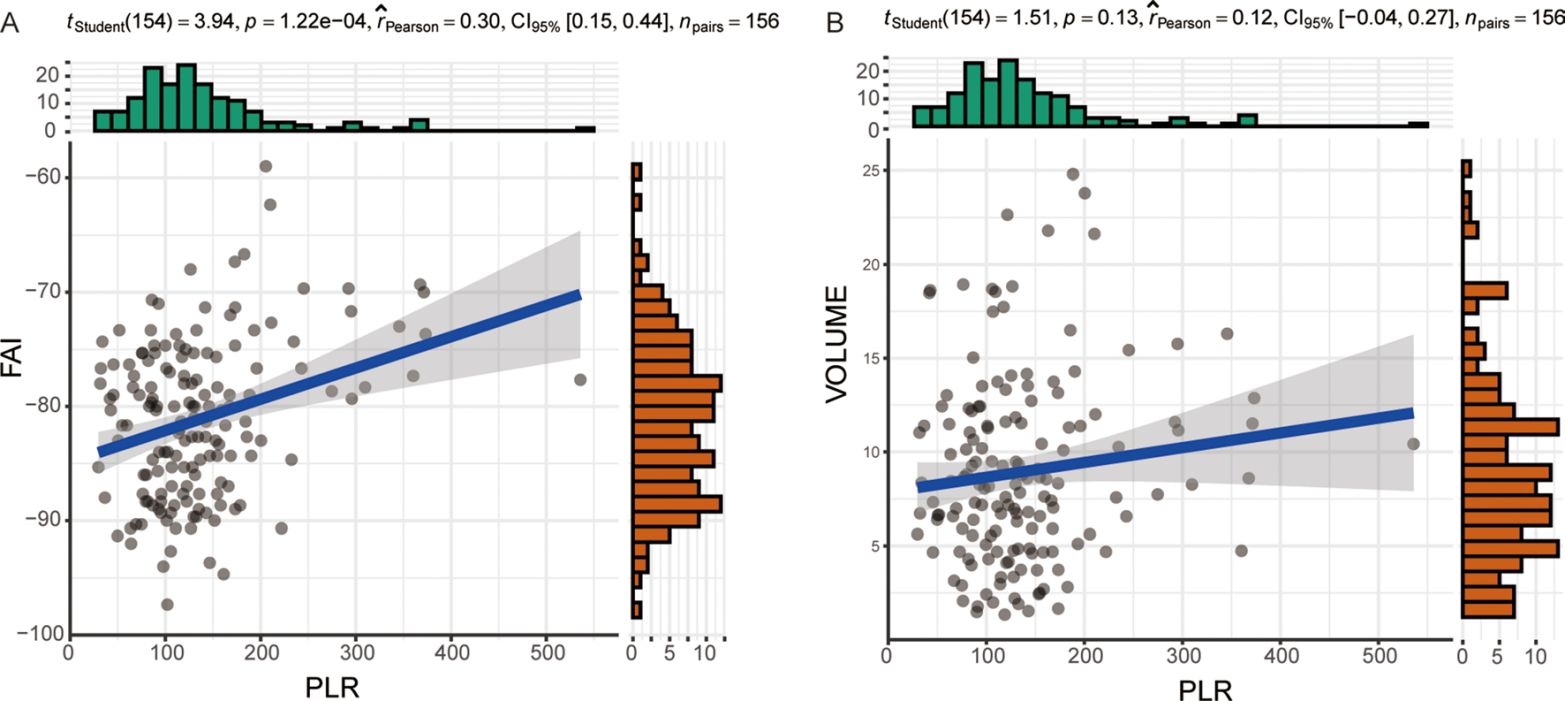
Correlation between PCAT-FAI and PCAT Volume with PLR. (**A**) PCAT-FAI is moderately positively correlated with PLR; (**B**) There is no correlation between PCAT volume and PLR. PLR, platelet to lymphocyte ratio; PCAT, peri-coronary adipose tissue; FAI, fat Attenuation Index

In order to further clarify the relationship between PLR and PCAT-FAI, we used Pearson correlation coefficient analysis to compare the correlation between PLR and PCAT-FAI. It showed that PCAT-FAI is moderately positively correlated with PLR (r: 0.298; *p* < 0.01) (Fig. 3A). Similarly, according to the Pearson correlation coefficient, no correlation was seen between PCAT volume and PLR (r: 0.121; *p* = 0.132) (Fig. 3B).

### Univariable and multivariable analyses of patients with NSTEMI and SA

To determine the potential of PLR, PCAT volume, PCAT-FAI and other baseline characteristics in predicting risk of NSTEMI, univariable and multivariable logistic regression analyses were conducted. Upon multivariable analysis, high value of PLR, PCAT-FAI, and PCAT volume were found to be possibly independent risk factors for NSTEMI after adjusting for hypertension, DM and smoking (Table 2).

**Table 2:** Univariable and multivariable analyses between NSTEMI and SA

Characteristic	Univariable Analyses		Multivariable Analyses	
	Hazard ratio(95%Cl)	*p* value	Hazard ratio(95%Cl)	*p* value
Hypertension	0.90(0.27–2.21)	0.819	-	-
Diabetes mellitus	0.93(0.43-2.00)	0.845	-	-
Smoking	1.41(0.62–3.17)	0.410	-	-
PLR	1.01(1.01–1.02)	0.001	1.01(1.00-1.02)	0.022
PCAT volume	1.19(1.08–1.31)	< 0.001	1.24(1.10–1.40)	< 0.001
PCAT FAI	1.20(1.10–1.30)	< 0.001	1.22(1.10–1.35)	< 0.001

PLR, platelet to lymphocyte ratio; PCAT, peri-coronary adipose tissue; FAI, fat attenuation index; FAI, fat Attenuation Index

### ROC curve analysis

**Fig. 4:**
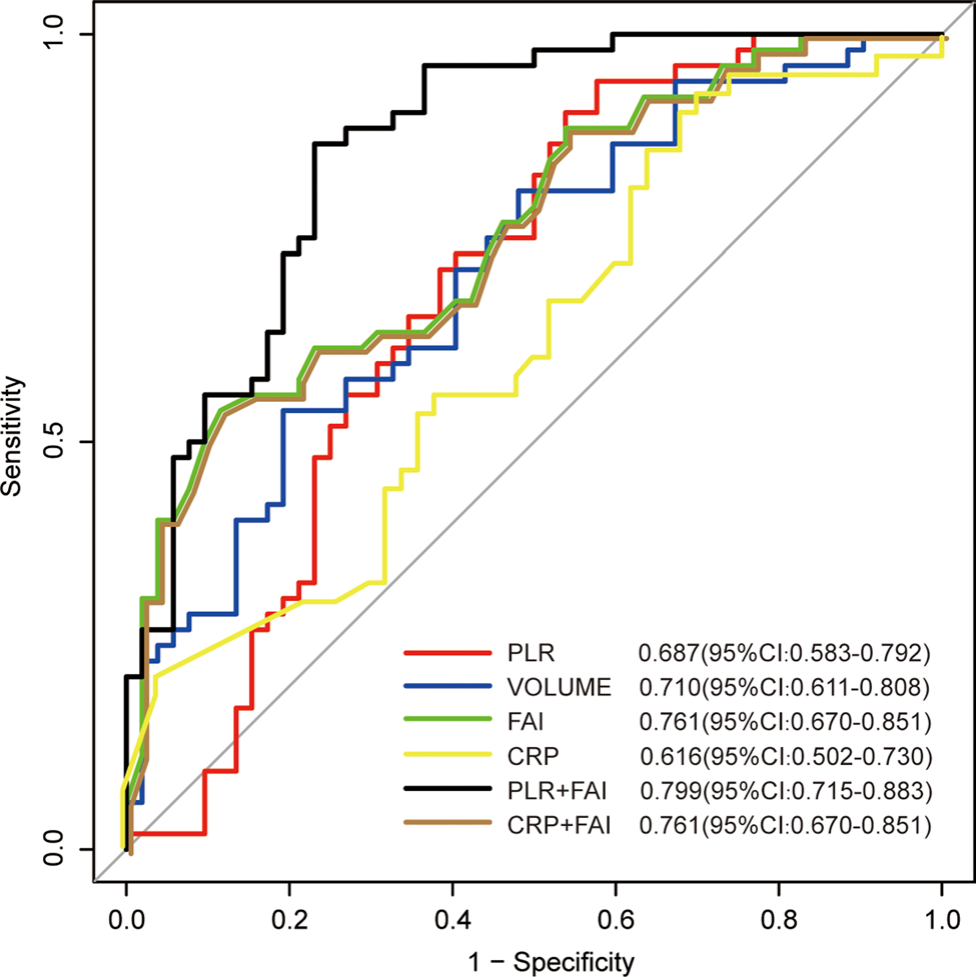
ROC Curve Analysis. PLR, platelet to lymphocyte ratio; PCAT, peri-coronary adipose tissue; FAI, fat Attenuation Index; CRP, c-reactive protein; ROC, receiver operating characteristic

In the analysis of ROC curves (Fig. 4), PCAT-FAI demonstrated an AUC of 0.761 (95% confidence interval [CI]: 0.670–0.851, *p* < 0.001) for predicting severe NSTEMI among CVD patients, slightly outperforming PLR (AUC 0.687, 95% CI 0.583–0.792, *p* < 0.001), CRP (AUC 0.616, 95% CI 0.502–0.730, *p* = 0.055), and PCAT volume (AUC 0.710, 95% CI 0.611–0.808, *p* < 0.001). Further evaluations indicated that a combination of PLR with PCAT-FAI yielded an AUC of 0.799 (95% CI 0.715–0.883) for NSTEMI prediction in patients with CVD, significantly surpassing the predictive capability of any single parameter. The optimal value of PCAT-FAI as an indicator for predicting the occurrence of NSTEMI was − 82.17HU, which yielded a sensitivity of 88.5% and a specificity of 53.8%. Similarly, PLR as an indicator for predicting the occurrence of NSTEMI was 170.8, which yielded a sensitivity of 46.2% and a specificity of 90.4%. Using PCAT-FAI in combination with PLR to identify NSTEMI achieved a sensitivity of 76.9% and a specificity of 70.2%.

## Discussion

In this study, we discovered that PCAT volume was elevated in NSTEMI patients compared to those with stable angina and non-CVD, while also observing higher PCAT volume in SA patients compared to non-CVD individuals. Additionally, we found that PCAT-FAI, a new marker of adipose tissue inflammation, was significantly elevated in NSTEMI patients compared to those with stable angina and non-CVD, with no significant difference between stable angina and non-CVD patients. Similarly, novel biomarker PLR was significantly higher in NSTEMI patients compared to stable angina and non-CVD patients, with no significant difference between stable angina and non-CVD individuals. In subsequent research, we compared the correlations between PLR, PCAT volume, and PCAT-FAI separately. The results revealed a moderate correlation between PLR with PCAT-FAI, while there was no correlation with PCAT volume.

In the pathophysiology of NSTEMI, the roles of lymphocytes and platelets are crucial [20, 21]. Lymphocytes have the ability to inhibit through various mechanisms [22–25]. They produce anti-inflammatory cytokines, promoting the resolution of inflammation [26]. Additionally, lymphocytes regulate the inflammatory response of macrophages and can prevent endothelial injury [27, 28]. However, in the process of atherosclerosis, prolonged inflammatory stimulation can impair lymphocyte function, causing them to lose their ability to effectively suppress inflammation [28]. This, in turn, promotes plaque rupture and thrombus formation [29–31]. On the other hand, upon the rupture of an atherosclerotic plaque, exposure of collagen and tissue factors triggers the activation of platelets [32–35]. These activated platelets then secrete cytokines, including platelet-activating factor (PAF), which facilitate the aggregation of more platelets, thereby recruiting and activating additional platelets [36, 37]. Following this, the activated platelets change shape and release granules filled with growth and clotting factors, aiding in the formation of a thrombus [38, 39]. In the context of NSTEMI, the inflammation and ensuing activation of platelets may lead to a rise in platelet counts [18, 40]. PLR is determined by dividing the count of platelets by the count of lymphocytes. Earlier studies have indicated that ACS leads to a notable rise in PLR levels, positioning it as an independent predictor of adverse outcomes in acute myocardial infarction [41–44]. Our results demonstrate that NSTEMI patients have a significantly elevated PLR when compared to those with SA and individuals without any CVD. Multivariable analysis indicates that PLR might act as an independent risk factor for NSTEMI. This evidence strengthens the idea that the interaction between inflammation and thrombus formation plays a key role in the progression of NSTEMI.

Another metric of interest in this study is PCAT-FAI, a relatively new imaging biomarker that quantifies changes in the composition and characteristics of fat surrounding the coronary arteries. This index reflects the inflammation within the adipose tissue adjacent to the coronary arteries. Inflammatory cytokines and other mediators released by inflamed adipose tissue surrounding the coronary arteries can diffuse into the adjacent coronary artery wall, fostering the progression of atherosclerosis, increasing plaque vulnerability, and leading to plaque rupture [13, 45, 46]. The increase in PCAT-FAI indicates heightened inflammation and changes in fat composition within the peri-coronary adipose tissue, which are associated with increased risk of coronary artery plaque instability and rupture [47, 48]. Therefore, it can be used to identify the risk of acute coronary events, such as myocardial infarction. Previous research has shown that PCAT-FAI is significantly elevated in the culprit vessels of patients with acute myocardial infarction [49, 50]. Analysis of PCAT inflammation in post-infarction patients revealed that PCAT inflammatory response in the culprit coronary artery is stronger than in the non-culprit coronary arteries [51]. In this study, we found that PCAT-FAI levels were higher in NSTEMI patients compared to those with stable angina and non-coronary artery disease, suggesting that PCAT-FAI could be an independent risk factor for NSTEMI through multivariable logistic regression analysis. Additionally, we found that NSTEMI patients have higher PCAT volume compared to SA and non-CVD patients, which is consistent with the results reported in previous literature [13].

In this study, we explored for the first time the correlation between PLR and PCAT-FAI as well as PCAT volume. The results revealed a moderate correlation between PLR and PCAT-FAI in elderly, but no correlation with PCAT volume. These findings suggest that inflammation of the peri-coronary adipose tissue, rather than the volume of adipose tissue, may be a significant factor leading to coronary plaque rupture and further promoting thrombus formation within the coronary arteries in elderly.

Given the potential of peri-coronary adipose tissue inflammation to act as a catalyst for plaque rupture and subsequent thrombogenesis, leading ultimately to myocardial infarction, this study assessed the prognostic abilities of PCAT-FAI, PLR, and their combined utility in forecasting NSTEMI outcomes. Findings demonstrate that PCAT-FAI provides a superior positive predictive capability for NSTEMI, whereas PLR excels in its negative predictive capacity. When utilized together, they enhance both the sensitivity and specificity of diagnosis, presenting an elevated prognostic efficacy.

## Conclusion

This study highlights the significant roles of PCAT volume, PCAT-FAI, and PLR in the context of NSTEMI, underscoring their potential as diagnostic and prognostic markers. Elevated levels of PCAT-FAI and PLR in NSTEMI patients underscore the critical interplay between adipose tissue inflammation and thrombosis in coronary disease progression. The study reveals that assessing PCAT-FAI and PLR together enhances diagnostic accuracy and prognostic prediction for NSTEMI, offering valuable insights for targeted therapeutic strategies.

### Limitation

Firstly, this study is a cross-sectional analysis, so the results indicate only a correlation between PCAT-FAI, PLR, and NSTEMI, without establishing causality. Secondly, retrospective studies are inherently susceptible to selection bias due to their fundamental design characteristics. Thirdly, as a single-center study, the findings have not been validated in other research centers. Fourthly, due to the sample size constraints in this study, we were unable to adjust for more confounding factors in our analyses. Finally, this study focuses solely on the relationship between PCAT and NSTEMI, without exploring the associations between other fat depots and NSTEMI.

## Data Availability

The datasets used and analyzed during the current study are available from the corresponding author on reasonable request.

## Abbreviations

PCAT: peri-coronary adipose tissue
FAI: fat attenuation index
PLR: platelet-to-lymphocyte ratio
NSTEMI: non-ST elevation myocardial infarction
STEMI: ST-elevation myocardial infarction
ECG: electrocardiography
CVD: cardiovascular diseases
CRP: cytokine C-reactive protein
IL: 6-interleukin-6
CT: computed tomography
CCTA: coronary computed tomography angiography
CAD: coronary artery disease
HRP: high-risk plaques
ACS: acute coronary syndrome
SA: stable angina
CAG: coronary angiography
PSM: propensity score matching
FOV: field of view
BMI: body mass index
ROI: region of interest
LAD: left anterior descending artery
LCX: left circumflex artery
RCA: right coronary artery
ANOVA: analysis of variance
DM: diabetes mellitus
ROC: receiver operating characteristic
CI: confidence interval
PAF: platelet-activating factor
PCI: percutaneous coronary intervention
